# Implementation of disaster medicine education in German medical schools – a nationwide survey

**DOI:** 10.3205/zma001475

**Published:** 2021-04-15

**Authors:** Nils Kasselmann, Christian Willy, Bernd D. Domres, Robert Wunderlich, David A. Back

**Affiliations:** 1 Bundeswehr Hospital Berlin, Clinic of Traumatology and Orthopedics, Septic and Reconstructive Surgery, Berlin, Germany; 2 Foundation of the German Institute for Disaster Medicine,Tübingen,Germany; 3 University Hospital Tübingen, University Department of Anesthesiology and Intensive Care Medicine, Tübingen, Germany; 4 Charité - Universitätsmedizin Berlin, Dieter Scheffner Center for Medical Education and Research, Berlin, Germany

**Keywords:** disaster medicine, students, medical schools, education, survey, Germany

## Abstract

**Objectives: **Floods, earthquakes and terror attacks in recent years emphasize the importance of disaster preparedness for the medical community. To best prepare doctors for providing optimal care in disaster situations, specific education and training should start at the medical school level. This study containes an online survey among German medical schools to evaluate the status quo of teaching disaster medicine and to reveal potential obstacles.

**Methods: **The dean’s offices of 36 German medical schools were contacted from April 2016 to May 2017. Via an e-mail link, recipients could anonymously and voluntarily access an online questionnaire (74 items, 42 with a four-point “Likert-like” scale, 12 yes/no questions and 20 with listed items to choose from). The answers were analyzed by descriptive statistics.

**Results: **A total of 25 medical schools participated in the survey. Twenty respondents were in favor of expanding disaster medicine teaching at their institutions. Incorporating single topics ranging from triage (n=21) to accidents involving radioactive materials (n=4) into the curriculum varied widely. Only two schools had established a teaching coordinator for disaster medicine and only one e-learning course had been established. Twenty-one respondents regarded funding issues and 18 regarded organizational matters to be major hurdles in the future.

**Conclusion: **Though most faculty representatives indicated that they favor expanding and implementing disaster medicine education, German medical schools still have a lot of room for enhancement in this field. The incorporation of e-learning tools could facilitate the expansion of disaster medicine teaching while simultaneously addressing the expressed concerns of the survey’s participants and guarantee nationwide standardization.

## Zusammenfassung

**Ziel: **Die Vorbereitung von medizinischem Personal auf Katastrophensituationen hat mit der steigenden Zahl von Naturkatastrophen und Terrorattacken an Bedeutung gewonnen. Um in der Ausbildung von Ärzt:innen einheitliche Grundlagen zu schaffen, muss bereits im Medizinstudium mit einer katastrophenmedizinischen Ausbildung begonnen werden. Im Rahmen einer Online-Umfrage an den Medizinischen Fakultäten in Deutschland wurde der Status quo dieser Ausbildung evaluiert, auch um etwaige Hindernisse aufzuzeigen.

**Methodik:** Die Prodekanate von 36 deutschen Medizinischen Fakultäten wurden von April 2016 bis Mai 2017 über einen Link via E-Mail zur Teilnahme eingeladen. Der Online-Fragebogen (74 Fragen, 42 mit Vier-Punkt Likert-ähnlicher Skala, 12 Ja/Nein Fragen und 20 Fragen mit Auswahlmöglichkeiten) konnte freiwillig und anonym beantwortet werden. Die Anworten wurden mittels deskriptiver Statistik ausgewertet.

**Ergebnisse:** 25 Fakultäten beantworteten die Umfrage. 20 Teilnehmer:innen befürworteten einen Ausbau der Lehre der Katastrophenmedizin. Die Einbindung von einzelnen Unterthemen variierte stark (Triage n= 21, Unfälle mit radioaktiven Materialien n = 4). Nur zwei Fakultäten hatten eine*n Lehrkoordinator*in für Katastrophenmedizin etabliert, nur eine bietet einen Onlinekurs an. Die Teilnehmer*innen erwarteten vor allem finanzielle (n=21) und organisatorische Hürden (n=18).

**Zusammenfassung: **Die meisten Fakultäten zeigten sich gegenüber einem Ausbau der katastrophenmedizinischen Lehre offen. Trotzdem gibt es viel Raum, die Lehre zu verbessern und das Angebot zu erweitern. Um eine nationale Standardisierung zu ermöglichen und die erwarteten Hürden zu adressieren, sollte vermehrt auf e-Learning Angebote zurückgegriffen werden.

## 1. Introduction

Global pandemics like the recent COVID-19 outbreak in 2020, or natural or technological disasters create major structural and logistical challenges for local and national health systems [1], [2], [3]. Various health care leaders have stated the importance of disaster medical training for all health care professions [3]. Consequently a substantial effort has been made to translate lessons learned from military experiences to civilian situations, e.g. tourniquet application during the Boston Marathon bombing [4], common civil-military training as reported for France [3], or introducing educational concepts such as a “terror and disaster surgical care” course in Germany [https://www.dgu-online.de/bildung/fortbildung/tdscr.html]. While the importance of disaster preparedness is undisputed, many studies identified substantial deficits in physician readiness across all medical specialties [5], [6], [7], [8]. Data from the United States of America (USA) showed that even residency programs for emergency physicians struggled to provide standardized and sufficient disaster preparedness training [9].

One approach to enhance disaster medicine knowledge in the medical community is to establish such training in medical schools’ undergraduate curricula. Incorporating such training should contribute to the required knowledge and skills for every future doctor, as recommended by the American Association of Medical Colleges in 2003 [10], following recommendations from the World Association for Disaster and Emergency Medicine (WADEM) [11].

In Germany, a *concept curriculum for disaster medicine in student education at German universities* was developed in 2006 by the federal ministry of internal affairs, the German society for disaster medicine and the federal office for civil protection and disaster relief. This concept was recommended for implementation at all medical schools by the *German Association of Medical Faculties* in 2007 [12]. In 2015, disaster medicine was also included in the *German National Competence-Based Learning Objectives Catalogue for Medicine* (NKLM) [http://www.nklm.de]. According to a master plan for medical studies 2020 [13], the NKLM aspires to be the basis of curriculum design in all German universities, requiring all medical students to build knowledge in disaster medicine. However, in 2015, a nationwide survey of 992 German medical students showed that disaster medicine in medical school curricula was still underrepresented and characterized by large institutional differences [14]. Similar results could also be shown among Dutch medical students, who had major gaps in knowledge and a low confidence in their own disaster medicine education [15]. 

But there are also positive examples in support of disaster medicine teaching in medical schools. In Italy, 37 medical schools were able to implement a national blended-learning curriculum for disaster medicine and 2,500 students were trained by student-led courses and e-learning methods over the course of six years [16], [17]. In the USA, a variety of courses have already been implemented with only a few of them mandatory, and with great differences in duration and content [10], [18], [19], [20], [21], [22], [23], [24]. In Germany, some innovative and successful disaster medicine-specific teaching offerings have been reported [12], [25], [26], [27]. However, these courses were only offered at individual medical schools – not nationwide - and thus could only reach a rather small fraction of all medical students in Germany. 

### 1.1. Study objective

Given the positive attitude of medical students towards the topic of disaster medicine, this study will identify the state of teaching in this field as well as potential obstacles for incorporating more offerings from the perspective of faculty leadership of 36 German medical schools, asking:

1. How widely has the concept curriculum been adopted in medical university curricula?
2. What hinders the expansion of disaster medicine teaching from a faculty point of view?
3. How can medical schools advance disaster medicine teaching in the future?

## 2. Methods

### 2.1. Designing the questionnaire

The initial questionnaire was created based on extensive literature research and validated through a peer review approach in 2016. Experienced professionals in the field of disaster medicine were asked to participate in the development phase of the questionnaire. The participants’ comments were collected, and the questionnaire adjusted accordingly. The survey followed the ethical directives of the Helsinki declaration. The final questionnaire for this study contained a total of 74 items, 42 with a four-point “Likert-like” scale, 12 yes or no questions and 20 with listed items to choose from. Additionally, the respondents were given the opportunity to add a comment after every question. The survey was then created in the online program SurveyMonkey^®^ (SurveyMonkey, Oregon, USA). By electronic pre-determination of the response options supplied by SurveyMonkey^®^, users were also offered the option of skipping or omitting certain questions. Access to the questionnaire was provided by a link, that could be integrated into e-mails.

#### 2.2. Content of the survey

**Figure 1:**
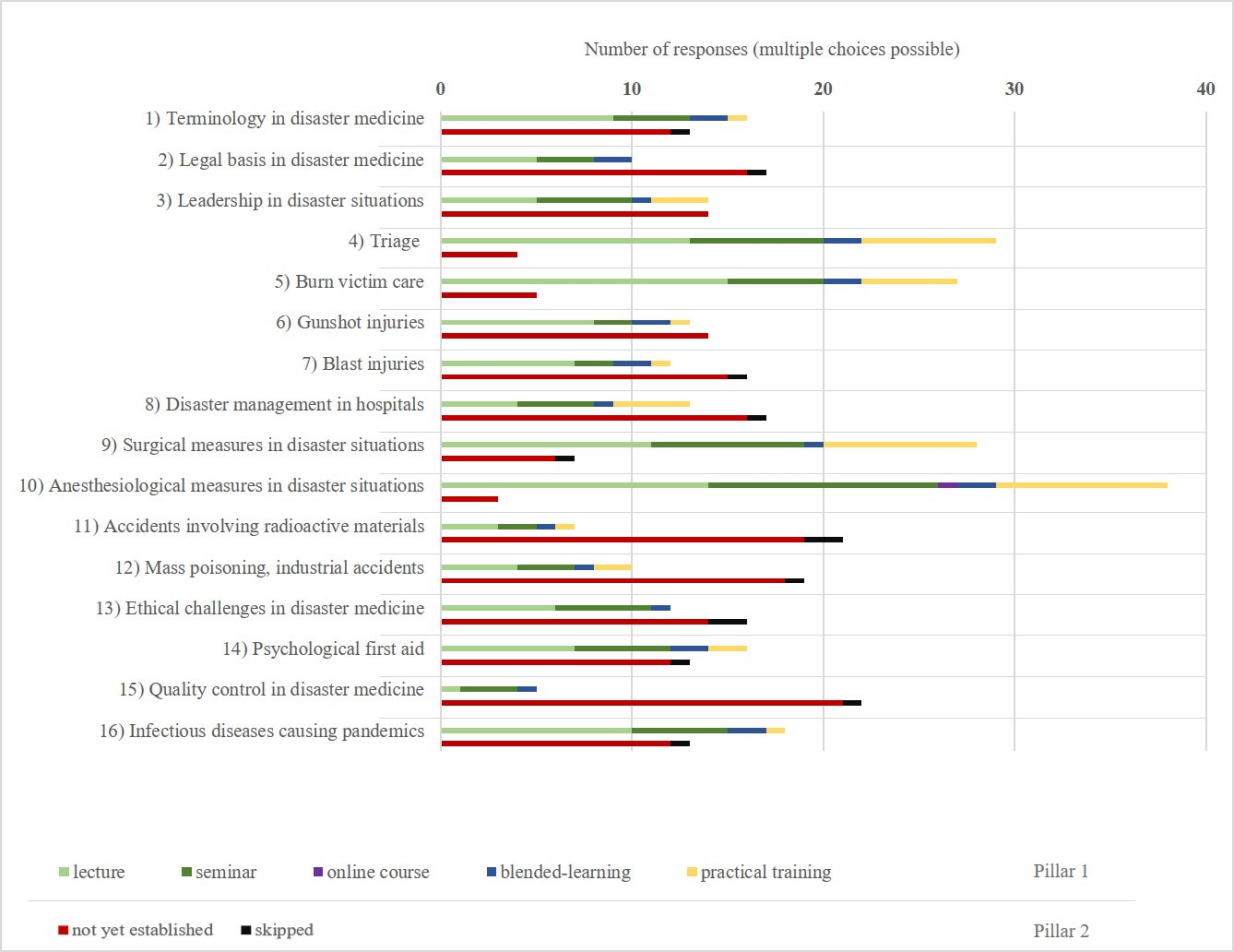
Overview over the existing teaching formats used to teach disaster medicine (multiple choices possible) and also the not established subcategories at the surveyed universities (n=25)

**Figure 2:**
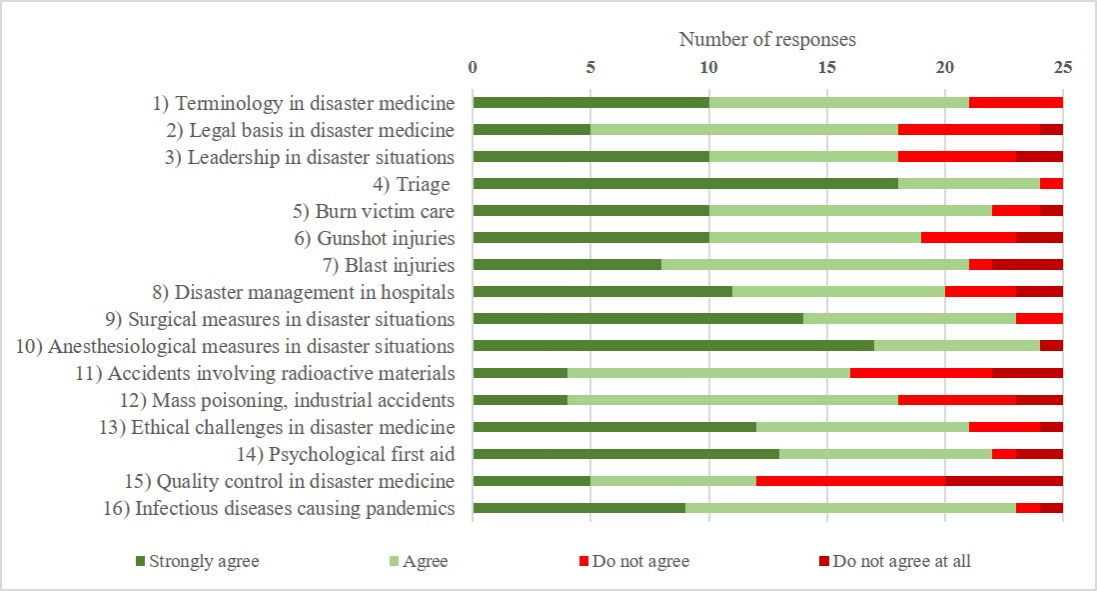
Votes for an enhanced integration into a medical curriculum of subtopics (4-point Likert-like scale) (n=25)

The questionnaire was based on the literature and the concept curriculum for disaster medicine and divided into the following subcategories:

1. *Respondents’ information* *(2 questions, 1x listed items, 1x Likert-like Scale):* Current position at the university and if they had encountered aspects of disaster medicine.
2. *Expansion of disaster medicine and the concept curriculum for disaster medicine (5 questions, 5x Likert-like Scale):* Questions about the sensibility of expansion of disaster medicine teaching in general; rating if the concept curriculum was known at their respected faculty, if it was actively used for curricular planning, if it was regarded as a good tool for standardization and if they tried to implement the curriculum as thoroughly as possible.
3. *Status quo of teaching disaster medicine (46 questions, 19x listed items, 21x Likert-like Scale, 6x yes/no): *16 subtopics were chosen according to the concept curriculum and the feedback of experts in the field for better characterization of teaching contents (see figure 1 (Fig. 1) and figure 2 (Fig. 2)). Subtopics 1, 2, 3, 4, 8, 11, 12, 13, 15, 16 (group 1) were graded as specific to disaster medicine alone. Subtopics 5, 6, 7, 9, 10, 14 (group 2) were graded as more general topics overlapping disaster medicine with other medical specialties. Questions about available teaching methods, agreement on further implementing the subtopics, when and how long disaster medicine was being taught, which departments were involved and what outside organizations collaborations had been established, were asked.
4. *Options for enhancing teaching in disaster medicine (21 questions, 15x Likert-like Scale, 6x yes/no): *This section included questions about preferred teaching methods; which schools had already established administrative structures (e.g. department chair, teaching supervisor) to support disaster medicine and which schools would consider establishing such structures in the future; what barriers to the expansion were identified and consideration to incorporate externally-developed e-learning materials.

#### 2.3. Performance and analysis of the survey 

The online survey link was sent out to the dean’s offices of 36 medical schools in Germany. The participation was voluntary and anonymous. The invitation asked for a member of the faculty with full oversight of the curriculum to answer the questionnaire. Preferably, respondents had already encountered disaster medicine situations in their careers. In order to increase the response rate, the dean’s offices were contacted between April 2016 to May 2017, sometimes repeatedly, by e-mail and telephone. Altogether, the survey lasted from April of 2016 to August of 2017. Final data was transferred from SurveyMonkey^®^ to Microsoft Excel^®^ Version 2013 (Microsoft Corporation, Redmond, WA, USA) and analyzed using descriptive statistics. The responses were evaluated as simple percentages using 25 (the number of respondents to the survey) as denominator. 

## 3. Results

In total, 25 out of 36 (70%) medical schools responded to the survey. 

### 3.1. Respondents’ information

Twenty-four of 25 (96%) respondents provided information on their current position at the university. Seven (28%) were medical doctors, eight (32%) were medical doctors with direct responsibilities in the field of disaster medicine, five (20%) were medical doctors with administrative responsibilities and four (16%) were university employees without a medical degree. Fourteen (56%) respondents encountered disaster medicine scenarios very often or often during their professional careers. Ten respondents (40%) reported that they had rarely encountered disaster medicine scenarios in their career, one (4%) respondent never encountered a need to practice disaster medicine. In the comments section of the surey, eight respondents (32%) described their personal experiences with disaster medicine. They reported multiple years of experience as emergency physicians, experience organizing mass casualty drills and experience with disaster planning for their medical schools. 

#### 3.2. Expansion of disaster medicine and the concept curriculum for disaster medicine

**Figure 3:**
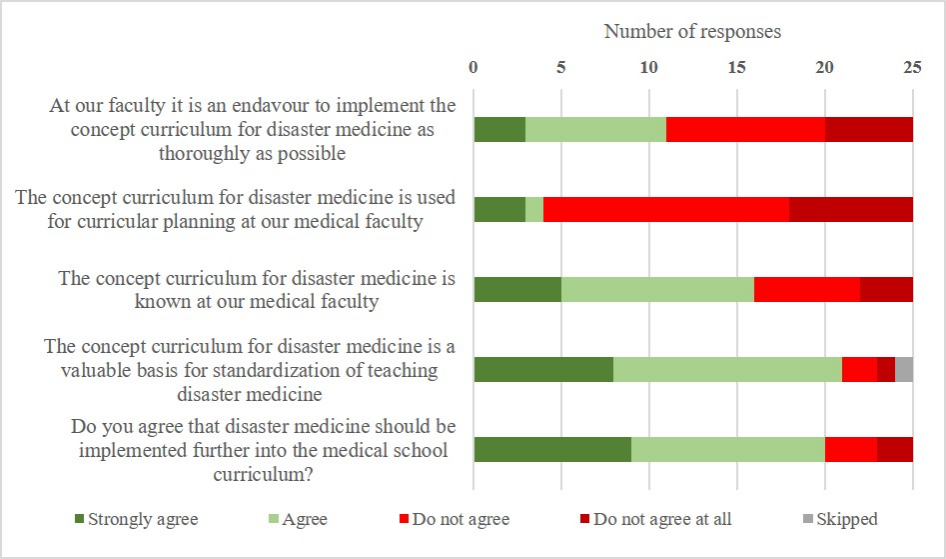
Implementation and expansion of disaster medicine teaching at German medical schools (4-point Likert-like scale) (n=25)

In total, 20 respondents (80%) agreed that disaster medicine should be incorporated further into the medical school curriculum, but in the comment section, some respondents (12%) remarked on the tension between the already mounting workload for medical students and the urgency of implementing disaster medicine. Most medical schools were aware of the concept curriculum’s existence (64%) but only four (16%) schools used it for curricular planning. Twenty-one (84%) respondents agreed that the concept curriculum was a valuable tool for nationwide standardization. Only 11 schools (44%) tried to implement the concept curriculum as thoroughly as possible (see figure 3 (Fig. 3)). 

#### 3.3. Status quo of teaching disaster medicine

As shown in figure 1 (Fig. 1), the respondents stated whether a subtopic of the concept curriculum was already implemented, and which teaching formats may have been utilized at their medical school. Skipped was considered not established. 

The data shows that on average, 59% of participating schools had not established the subtopcis specific to disaster medicine (group 1). Moreover, 37% of schools had not regularly addressed the more general subtopics (group 2).

Topics concerning chemical, biological, radioactive and nuclear (CBRN) threats were only taught in a few universities (11: accidents involving radioactive materials, 16%; 12: mass poisoning, industrial accidents, 24%). Triage was taught in most medical schools (84%). 

Overall, lectures and seminars were the most utilized teaching formats. Practical training was mainly offered for surgical (32%) and anesthesiologic (36%) measures in disaster situations. Seven universities offered practical triage training (28%). Only one (4%) university had established an online course. 

The participants were then asked whether they supported expanding disaster medicine teaching regarding the given subtopics (see figure 2 (Fig. 2)). There was a strong majority in favor of every subtopic, except for *quality control in disaster medicine* (48%). 24 out of 25 (96%) participants agreed that learning about triage was important for every medical student. There were also strong majorities in favor of teaching students about gunshot injuries (76%) and blast injuries (84%). 

According to the surveyed institutions, many began teaching aspects of disaster medicine during the fifth semester (32%). Only two schools started teaching disaster medicine earlier. Disaster medicine was mostly taught for one (20%) or two (24%) semesters. 

With disaster medicine not being its own specialty in Germany, the medical schools were asked to identify the medical disciplines primarily responsible for disaster medicine education. Anesthesiology was involved in 21 (84%) and trauma surgery in 18 (72%) of the medical schools. Other specialty fields were more rarely involved. 

When asked whether they had established long-standing collaborations with organizations associated with disaster relief, only three schools (12%) answered that they cooperated with the German society for disaster medicine. Thirteen universities (52%) cooperated with civilian nonprofit organizations, mainly for teaching first aid. Only one university cooperated with the medical services of the Bundeswehr. One respondent answered in the comment section that they had soldiers lecturing on the topic of civil-military collaboration. Three medical schools cooperated with other disaster relief organizations. In the comment section, respondents primarily referred to collaborations with local fire departments, providing instruction in teaching first aid. 

#### 3.4. Options for enhancing teaching in disaster medicine

**Figure 4:**
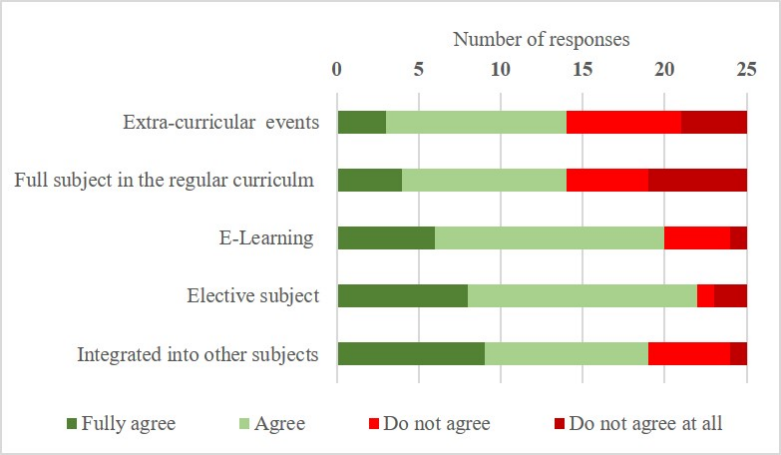
Most appropriate teaching formats for disaster medicine (4-point Likert-like scale, multiple answers possible) (n=25)

The respondents were asked where they felt the best settings were to implement disaster medicine teaching. Fourteen (56%) were in favor of establishing disaster medicine as its own teaching field within the regular curriculum, while an elective module was most strongly supported (88%). Only five (20%) schools did not agree that e-learning is suitable for disaster medicine (see figure 4 (Fig. 4)). No medical schools reported a “chair” for disaster medicine in Germany, but one medical school reported establishing an “institute” for disaster medicine. Two medical schools (8%) had already established a teaching supervisor for disaster medicine. Sixteen institutions (64%) were in favor of establishing a teaching supervisor and seven schools (28%) saw the need for a chair in the field of disaster medicine. 

**Figure 5:**
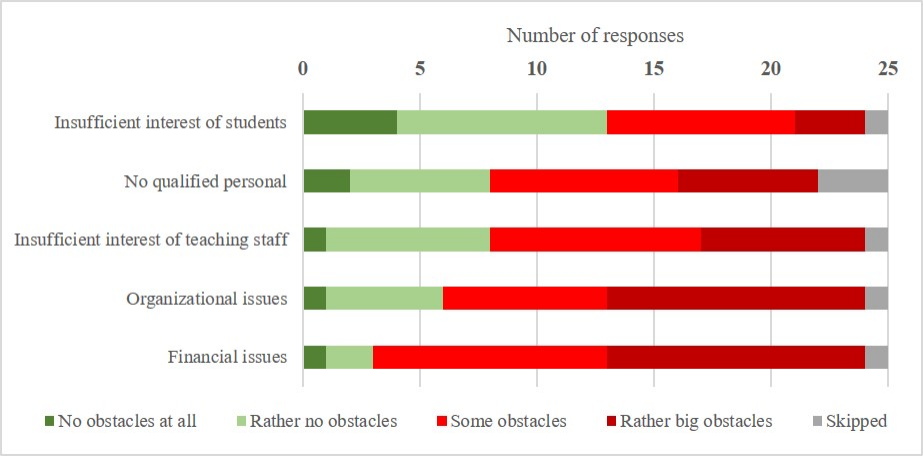
Expected obstacles for the expansion of disaster medicine teaching in Germany (4-point Likert-like scale) (n=25)

Figure 5 (Fig. 5) displays the respondents’ opinions about possible obstacles in the path for the advancement of disaster medicine. Financial challenges were regarded as an obstacle by a majority of 21 (84%) schools. Sixteen (64%) schools believed that there would be insufficient interest among teachers. 

## 4. Discussion

Disaster medicine education at medical schools still faces many challenges despite wide agreement of its necessity [3], [12], [15]. From a global perspective, efforts for the advancement of disaster medicine teaching vary greatly and depend on individual efforts [16], [22], [24], [28]. In Germany, a *concept curriculum* from 2006, recommended for voluntary use at every medical school by the *German Association of Medical Faculties*, could serve as an excellent foundation for implementing and enhancing teaching offerings [15], [27], [29]. Despite the recommendation to voluntarily adopt the *concept curriculum* in 2007, German medical students still recently reported insufficient training in disaster medicine [14].

This survey was therefore conducted to accurately evaluate the status of disaster medicine education in Germany from the perspective of the medical schools to identify potential obstacles and make recommendations accordingly. 

Concerning the respondents’ information, contacting the schools via the dean’s office ensured that the correct individual respondents were able to represent their respective medical school. Most respondents were medical doctors with clinical or administative responsibilities and more than half reported experience with disaster medicine throughout their careers. Consequently, it would appear that the approach to the medical schools through the dean’s office resulted in well-qualified respondents completing the survey. 

It is encouraging to note that 80% of the responding medical schools were in favor of expanding disaster medicine teaching, aligning with students’ opinions in every major survey published to date [14], [15], [27], [29], [30]. Nevertheless, the *concept curriculum* was utilized only by four schools for actual curricular planning and nine participants did not even know of its existence. Despite these obvious shortcomings, most schools agreed that the *concept curriculum* could be a good means of standardization. The discrepancy between the *concept curriculum’s* acceptance and its lack of visibility could be a major reason for the slow process of implementation. In the future, a quality control process could be implemented for curricular innovations in disaster medicine to ensure a sustainable change in this discipline. 

The state of disaster medicine education in Germany is highly concerning. The above mentioned apparent deviation between theory and practice seems to be represented in the high variety in content, duration and form of offered courses [25], [26], [31]. Although the existing courses in Germany are reported to be based on the *concept curriculum*, they are still highly variant and do not represent the full content recommended in the curriculum. Moreover, all of the courses offered were either electives or extra-curricular so it might be assumed that students participating in the courses had a prior interest in disaster medicine.

Regarding the German *concept curriculum* for disaster medicine, the survey revealed that topics especially related to the disciplines of anesthesiology and surgery were recognized by the medical schools and thus subtopics interconnecting disaster medicine with other medical specialties. Other topics such as developing leadership skills in disaster events, industrial accidents, mass poisoning, chemical/biological/radiological/nuclear (CRBN) accidents and practicing disaster management in hospitals were largely ignored. These discrepancies might be explained by the fact that faculty competencies in the former were not sufficiently represented in the participating medical schools. This also seems to be the reason of the widely-accepted practice to integrate disaster medicine into other specialties. The collected data clearly shows the need for a specific disaster medicine framework, so topics covered solely by disaster medicine will not be left out. 

When asked to identify a suitable format to teach and enhance disaster medicine education, elective coursework was preferred by 88% of the medical schools, while only 56% were in favor of establishing it as its own field of study and teaching. However, it has been shown that while electives are highly accepted by students, electives are only able to reach a few students and can therefore only be a temporary remedy [25], [32], [33]. This rather cautious attitude towards engagement in enhancing disaster medicine teaching may also be explained from the responses that most medical schools expected financial and administrative obstacles when expanding disaster medicine teaching. Fourteen respondents were worried that there may be a shortage of skilled teaching personnel. 

It can be suspected that without a departmental representation and also a professorship for disaster medicine, the disaster medicine community’s voice may be diluted in an environment of voluntary implementation. With only two schools having established a teaching coordinator for disaster medicine, creating and filling this position at more medical schools could be the first step to lead future efforts with the goal of establishing a chair for disaster medicine at multiple universities. To the best of the authors’ knowledge, there is no evidence of disinterest of students suspected by some of the surveyed medical schools. The disaster medicine community consequently has to increase its effort to communicate the students’ high interest as a key resource to further education.

A weakness of this study is that 11 of the 36 contacted medical schools did not respond. Furthermore, it was conducted in only one country and therefore the results might not be directly comparable to other countries with different educational systems. The respondents were chosen by their medical school, but there was no control mechanism in place to ensure the respondents had a full overview of the curriculum and completed the questionnaire thoroughly. Because disaster medicine concepts are not consolidated in one class but throughout different classes, some of the collected data might not be 100% accurate due to a lack of oversight. The questionnaire was not created using a DELPHI method but based on expert opinions.

It can be concluded that after more than 10 years in existence, the quasi-voluntary implementation of the *concept curriculum* has been slow and difficult. This could indicate that a change in strategy is necessary to achieve wide-spread acceptance, even though the survey showed that most administrators are in favor of expanding disaster medicine teaching. A common strategy in Germany seems to be lacking. 

Disaster medicine advocates all over the world are arriving at new and innovative methods to incorporate the topic into a medical school´s curricula but so far, their efforts have only reached a small fraction of students [17], [24], [25]. Real change will only be achieved through a coordinated, nationwide approach, comparable to the Italian efforts previously mentioned that included 37 medical schools [17]. 

This approach could also address the financial and organizational concerns of medical schools by establishing clear responsibilities and delineated lines of authority that will facilitate adoption of the topic at the specific medical schools. With special regards to the current COVID-19 pandemic, the incorporation of the key knowledge content into a strong e-learning component might also meet current needs, based on the German *concept curriculum*. Concerning digital solutions, a solid base for the implementation of e-learning tools in disaster medicine education has already been reported [34], [35]. The *concept curriculum for disaster medicine* could be structured into an open-source e-learning course available to all medical faculties, freeing up time for more practical training and local alterations. Nearly all medical schools in Germany are using an online learning management system and as shown in the results of this survey, these schools are willing to present learning materials developed outside their own school [36]. This approach could enhance the exchange of existing offerings and assist in the future development of evidence-based teaching modules that adhere to the official recommendations. 

If e-learning for disaster medicine is provided within courses primarily covering other specialties, there is the danger of marginalization. Therefore, e-learning should be made available in a blended-learning format to deepen knowledge and include practical skill training specific to disaster medicine.

While this solution does not address the issue of too few qualified teaching staff, it would assist less-specialized personnel and support more structured teaching efforts. 

A minority of universities had established a partnership with external organizations concerning disaster medicine teaching. Deepening such partnerships could aid in the execution of practical skills trainings and mobilize existing expertise in disaster relief, as it has been shown in Berlin and Tuebingen [25], [26], [27]. The realization of such training efforts could include a skill *parkour* or even computer-based simulations, which can be customized locally. The development could be coordinated by different organizations such as the German society for disaster medicine, the German society for orthopedics and traumatology, the German society of anesthesiology and intensive care medicine or also the armed forces medical service in a close exchange with the medical schools. As this development progresses, it would be appropriate to evaluate the *concept curriculum* for its relevance to current challenges in the medical field. For example, the COVID-19 pandemic could highlight a need for a stronger focus on infectious diseases, their transmission, prevention and “community-spread”. 

## 5. Conclusions

This survey showed a high acceptance of the idea to enhance disaster medicine teaching by German medical school officials. Nevertheless, the overall implementation to date has been slow and highly variant. The *concept curriculum* is rarely used for curricular planning, revealing a lack of a coordinated approach. Subtopics not overlapping with the fields of surgery and anesthesiology are often overlooked, suggesting that there is no clear advocate for the advancement of disaster medicine education. Measured against the goal of complete adoption of the *concept curriculum*, the current strategy must be considered a failure. To guarantee a basic disaster medicine education for every medical student, a coordinated effort to establish a nationwide blended-learning curriculum to include the recommended time for face-to-face teaching and practical skills training within the regular curriculum should be established. Using this “coordinated approach”, concerns about financial and administrative issues could be mitigated, if not eliminated. This is the only way to prepare and implement solutions to address the growing threat of disasters and pandemics that might likely be faced now and by generations to come.

## Abbreviations

- CBRN – Chemical, Biological, Radiological and Nuclear
- NKLM – National Competence-Based Learning Objectives Catalogue for Medicine
- TDSC – Terror and Disaster Surgical Care
- USA – United States of America
- WADEM – World Association for Disaster and Emergency Medicine

## Data

Data for this article are available from Dryad Digital Repository: http://doi.org/10.5061/dryad.x0k6djhgg [37]

## Competing interests

The authors declare that they have no competing interests.
